# Challenges for Relative Effectiveness Assessment and Early Access of Cancer Immunotherapies in Europe

**DOI:** 10.3389/fmed.2016.00056

**Published:** 2016-11-14

**Authors:** Mira Pavlovic

**Affiliations:** ^1^Medicines Development and Training Services, Paris, France; ^2^Department of Dermatology, Tenon Hospital, Paris, France

**Keywords:** cancer immunotherapy, endpoints, added benefit, relative effectiveness, early access to market

## Abstract

Clinical endpoints relevant for relative effectiveness assessment (REA) reflect how patients feel, function, or survive. Outcome data requested by health technology assessment (HTA) bodies in Europe to support reimbursement of an anticancer drug are based on final endpoints coming from completed comparative phase 3 trials; overall survival improvement is the preferred criterion for the demonstration of the patient benefit in this field. Recent arrival of new treatments that target identified functional genetic mutations (“targeted therapies”) or PD-1/PD-L1,2 axis (“immunotherapies”) and their combinations have profoundly changed treatment strategies in cancers as they considerably improve patient survival, but also raise new challenges in REA and decision-making process in Europe as compared to the REA of “classical” chemotherapies. In addition, recent regulatory initiatives to support accelerated clinical development and approval of innovative cancer immunotherapies based on non-final endpoints, such as priority medicines through the European Medicines Agency, represent an additional challenge for HTA bodies and decision makers. In order to support adequate data generation for REA of anticancer drugs and especially for drugs candidates for accelerated assessment and early access to market, a close and open dialog of all stakeholders involved in development of such drugs is crucial.

## Introduction

The choice of adequate endpoints as well as adequate comparator(s) in well-designed clinical trials is crucial to support both marketing authorization and reimbursement decisions. Clinical endpoints for relative effectiveness assessment (REA) considered relevant for patients measure morbidity, mortality, and health-related quality of life (HRQoL). In the field of oncology, overall survival (OS) data coming from comparative phase 3 trials are generally requested by health technology assessment (HTA) bodies to demonstrate patient benefit for an anticancer drug.

As compared to classical chemotherapy, cancer immunotherapies and their combinations raise many challenges in REA, even when based on endpoints considered relevant by HTA bodies. Recent regulatory initiatives to support accelerated clinical development and approval of innovative cancer immunotherapies, based on intermediate or surrogate endpoints, represent an additional challenge for HTA bodies and decision makers in Europe.

## REA of Anticancer Drugs – General Requirements

Whatever the disease, clinical endpoints for REA recognized as endpoints relevant for patients broadly measure mortality, morbidity (due to disease or its treatment), and HRQoL; they reflect how a patient feels, functions, or survives (1). This definition of clinical endpoints (2) is accepted by most reimbursement agencies in Europe (3). Simultaneous assessment of all relevant endpoints is a hallmark of REA; even if a trial is powered on a primary endpoint, the added clinical benefit of a new drug will be assessed on all endpoints relevant for a disease or its treatment (1). Preference is clearly given to long-term or final endpoints (3). Surrogate endpoints are accepted only when they are validated, i.e., when there is compelling evidence of a clear and consistent correlation between the effect of treatment on the surrogate and the effect on the final outcome of interest (4). In the field of oncology, endpoints generally recognized as relevant for patients are: OS improvement (final endpoint), improvement in progression-free survival, or survival without symptoms, time until the start of new treatment, the possibility to access curative alternative treatments (for example surgery or a new chemotherapy), improvement in key disease symptoms (intermediate endpoints), toxicity reduction, and improvement or lack of noticeable alteration of quality of life.

Overall survival improvement is the preferred criterion by HTA bodies in Europe for the demonstration of the benefit of an anticancer drug; globally, a survival gain of at least 2–3 months or more would be considered appropriate for a new drug versus an adequate comparator, even if there is no officially defined threshold (5). In cancers with low mortality, progression-free survival (PFS) and (exceptionally) response rates may be acceptable as intermediate endpoints.

The role of PFS role is double in this respect: as substitution endpoint or as endpoint with an intrinsic value in relation to quality of life and other clinical benefits (symptoms reduction) and as an intermediate clinical endpoint more or less correlated to OS when the latter is not available (5). The problem with using PFS as an intermediate and/or substitution endpoint is not specific to oncology, but it has a particular importance in cancers with long survivals. As intermediate endpoints tend to overestimate the medical benefit (6), even if validated for a particular tumor and stage of disease, OS is clearly preferred by reimbursement agencies (5).

The assessment of the added clinical benefit of a new drug is always a comparative one; it is based on its comparison to an adequate comparator or treatment strategy/ies defined by HTA bodies, by using appropriate clinical endpoints, relevant to the main characteristics of the disease/condition to treat, the target population and the aim of treatment.

Therefore, in the case of an irrelevant comparator, and/or a wrong choice of endpoints, or clinically irrelevant difference in OS, added benefit may not be granted. In the absence of OS data, lower added benefit may be granted if it is based on PFS data only. As mature data are requested whenever possible, interim analysis is generally not recommended, especially on PFS, but also on OS data if too premature.

As REA should support clinical practice guidelines, data to support the potential place of the product in the treatment pathway (treatment after progression, possibility/effectiveness of subsequent treatments) and effectiveness in relevant patient subpopulations (slowly progressing versus fast progressing patients) are also of interest.

## REA of Cancer Immunotherapies

As stated, outcome data requested by HTA bodies to support reimbursement of anticancer drugs are based on final (“hard”) endpoints coming from completed comparative phase 3 trials. In addition to OS (final endpoint), other patient-relevant endpoints are also assessed (such as time to symptomatic progression, symptoms, HRQoL).

The recent arrival of new treatments that target identified functional genetic mutations (“targeted therapies”) or PD-1/PD-L1,2 axis (“immunotherapies”) and their combinations have profoundly changed treatment strategies in cancers as they considerably improve patient survival, but have also raised new challenges in REA and decision-making process in Europe as compared to the REA of “classical” chemotherapies. In addition, recent regulatory initiatives to support accelerated clinical development and approval of cancer immunotherapies, based on intermediate or surrogate endpoints such as PRIME through the EMA, represent an additional challenge for HTA bodies and decision makers.

## Targeted Therapies

If we review some recent developments and REAs of targeted monotherapies and their combinations in Europe, we shall find the following common features: enriched study designs, aimed to show superiority, versus reference treatment (chemotherapy for targeted monotherapies and targeted monotherapy for combination targeted therapies) on intermediate and final endpoints, high response rates, relevant PFS and OS improvements and acceptable (or decreased) toxicity (with two targeted drug combinations). Both product development programs and study results do not represent a real challenge for REA and reimbursement: binary reasoning (presence or absence of mutation), validated companion test, existence of regulatory and HTA guidelines on drug-biomarker co-development and assessment, clear results, and lack of difficulty understanding product effectiveness and safety profile, make REA rather straightforward.

## Immunotherapies

The situation is clearly different with current immunotherapies targeting the PD-1/PD-L1,2 axis. The evidence provided by developers raises challenges at each step of REA: choice of adequate population, choice of adequate dose, and assessment of response to treatment. Indeed, despite the hypothesis that the expression of PD-L1 on immune and tumor cells is correlated with efficacy, different companies have chosen different approaches to define target population and support their product effectiveness for the same indication (e.g., non-small cell lung cancer): nivolumab was tested and approved in overall patient population irrespective of PD-1 status and pembrolizumab in PD-1 positive patients (50% cutoff) only. For combination immunotherapies, responses have been observed regardless of tumor expression of PD-L1 at baseline (ipilimumab + nivolumab, tremelimumab + durvalumab)(7).

With regards to the choice of adequate dose, the absence of a clear relationship between the dose and antitumor activity and toxicity has raised criticisms from the HTA bodies and difficulties in REA; the choice of dose remains an issue for further product development and use [e.g., further study requested by the food and drug administration (FDA) to compare ipilimumab 3 mg/kg – approved dose and 10 mg/kg Q3W – unapproved dose, tested in the adjuvant setting in melanoma]. The choice of dose(s) is even more difficult for combination immunotherapies (7), and may involve different scenarios, the most frequent being to administrate one product at fixed dose and the other at increasing doses and assess tumor growth inhibition.

Except for OS, the assessment of response to treatment to immunotherapies is not straightforward due to pseudo progressions and their impact on the assessment of intermediate effectiveness endpoints (timing of assessment, adaptation of existing tools, characterization of progression, and decision to allow for cross over or change treatment).

Low response rates, long duration of response, and long OS in some patients, all improved with combination immunotherapies but with much higher toxicity and high costs, render decision-making rather difficult both in curative and supposedly also in an adjuvant setting.

## Accelerated Assessment and Early Access to Market

As the first step to market access, a new medicinal product requires marketing authorization from a regulatory agency, based on its acceptable quality, safety, and efficacy in a given patient population, in comparison to placebo and/or (an) appropriate comparator(s) in pre-marketing clinical trials. The second step to market access to support a reimbursement decision is the REA that compares the benefits and harms of a new drug in a target population with one or more alternative interventions (e.g., standard of care), evaluating whether a new treatment has an added benefit or is equivalent to existing alternatives for achieving the desired results when provided under the usual circumstances of health-care practice.

Both marketing authorization issued by the EMA and positive reimbursement decisions issued by national authorities are necessary for market access of a new drug.

Current European pharmaceutical legislation includes different possibilities aimed at facilitating patients’ early access to new drugs that address public health needs, such as an accelerated assessment procedure, a conditional marketing authorization, and the possibility of a compassionate use opinion by the committee for human medicinal products (CHMP), defining at European level the criteria and conditions for the use of non-authorized medicinal products made available to patients through national patient access programs (prior to a marketing authorization).^1^

To optimize accelerated assessment, EMA has launched the PRIority Medicines (PRIME) program^2^ to support the development of innovative medicinal products (such as cancer immunotherapies and/or targeted therapies), which is supposed to have a major public health interest in conditions where there is unmet medical need. A major therapeutic advantage to patients should be demonstrated through a clinically meaningful improvement of efficacy, and/or an impact on the prevention, onset, or duration of the condition. Access to the PRIME scheme will depend on both the magnitude of the treatment effect, which could include duration of the effect, and the relevance of the observed clinical outcome.^3^^,^^4^

Early interaction between the applicant and multiple stakeholders, involving EMA, HTA bodies, and patients, on key decision points/issues for the preparation of marketing authorization application and reimbursement dossiers, is foreseen to ensure the generation of a robust data package and to facilitate timely access to patients. Existing scientific advice procedures (independent or integrated regulatory and HTA advice, either on the national or European level) may be used for this purpose (8).^5^

## Discussion

The improvement in OS remains a gold standard for REA to inform reimbursement decisions for an anticancer drug. Intermediate endpoints such as response rate, duration of response, and PFS are not easily accepted as predictors of clinical benefit and assessed (only) in the absence of OS data. Surrogacy of these endpoints to predict OS depends on tumor type and stage of disease, as well as on the therapeutic agent used. Delayed responses and pseudo-progressions on some immunotherapies make this task even more difficult. With the current HTA mind-set, limited development programs that might be sufficient for an accelerated assessment and regulatory approval might not be sufficient to support reimbursement. A recent survey on reimbursement status of medicines granted conditional approval shows that HTA decisions still remain independent on the regulatory route of approval and consider only data available to support REA and not the regulatory approval pathway (9).

It is supposed that the arrival of different drug combinations in the rapidly evolving field of cancer immunotherapies and better understanding of the synergy between different treatment modalities, relationships between responses to treatment, durations of response, toxicities and survival, will ultimately influence REA reasoning and reimbursement decisions. In addition, the establishment of the surrogacy between intermediate endpoints and OS might allow for earlier access to market based on non-final endpoints.

An open dialog of all stakeholders involved in the development of these products in order to support adequate data generation for early assessment and access to market is crucial in this respect. In general, the choice of adequate endpoints and comparators to support both (accelerated) assessment and (early) access to market is crucial in any drug development. The recent analyses of parallel EMA – HTA scientific advice with regard to requests for the trial population, study design, endpoints, and comparators, show an agreement, important for trial population and study design and rather weak for the choice of endpoints and comparator(s) (10)^6^; on the contrary, the agreement among HTA bodies was rather high on most items (10).

Despite divergences in regulatory and pricing/reimbursement requests for data generation, it is postulated that these can be integrated within global product development and outcomes for benefit-risk and REA, standardized in order to be able to “file the same and propose the same,” both for regulatory and reimbursement purposes.

This does not guarantee that a drug will be reimbursed – its reimbursement status will probably vary from one country to another. However, there is a hope that decreasing uncertainty of assessment will ultimately facilitate market access and patient care.

## Author Contributions

Dr. MP has entirely written the submitted article.

## Conflict of Interest Statement

The author declares that the research was conducted in the absence of any commercial or financial relationships that could be construed as a potential conflict of interest.

## Footnotes

^1^EMA/531801/2015. Development support and regulatory tools for early access to medicines. Available from: http://www.ema.europa.eu/docs/en_GB/document_library/Other/2016/03/WC500202631.pdf.

^2^EMA/89921/2016. Launch of PRIME – paving the way for promising medicines for patients. Available from: http://www.ema.europa.eu/docs/en_GB/document_library/Press_release/2016/03/WC500202669.pdf.

^3^EMA/191104/2015. European Medicines Agency Guidance for applicants seeking access to PRIME scheme. Available from: http://www.ema.europa.eu/docs/en_GB/document_library/Other/2016/03/WC500202630.pdf.

^4^EMA/CHMP/57760/2015. Enhanced early dialogue to facilitate accelerated assessment of PRIority MEdicines (PRIME). Available from: http://www.ema.europa.eu/docs/en_GB/document_library/Regulatory_and_procedural_guideline/2016/03/WC500202636.pdf.

^5^European Medicines Agency. Guidance for Applicants seeking scientific advice and protocol assistance EMA/691788/2010 Rev. 7. Available from: http://www.ema.europa.eu/docs/en_GB/document_library/Regulatory_and_procedural_guideline/2009/10/WC500004089.pdf.

^6^Available from: http://www.efpia.eu/uploads/Modules/Documents/150116-merck-ema-hta-analysis-final-report.pdf.

## Abbreviations

CHMP, Committee for Medicinal Products for Human Use; EMA, European Medicines Agency; HRQoL, health-related quality of life; HTA, health technology assessment; OS, overall survival; PFS, progression-free survival; PRIME, priority medicines; REA, relative effectiveness assessment.
